# Primary healthcare in the aftermath of the COVID-19 pandemic: a qualitative interview study in Sweden

**DOI:** 10.1136/bmjopen-2024-085527

**Published:** 2024-07-27

**Authors:** Hanna Fernemark, Maria Hårdstedt, Janna Skagerström, Ida Seing, Elin Karlsson, Per Nilsen, Kristina Görel Ingegerd Schildmeijer

**Affiliations:** 1 Department of Health, Medicine and Caring Sciences, Linköping University, Linköping, Sweden; 2 Primary Health Care Center, Lambohov, Region Östergötland, Linköping, Sweden; 3 Vansbro Primary Health Care Center, Vansbro, Sweden; 4 Center for Clinical Research Dalarna, Uppsala University, Falun, Sweden; 5 Research and Development Unit, Region Östergötland, Linköping, Sweden; 6 Department of Behavioural Sciences and Learning, Linköping University, Linköping, Sweden; 7 Halmstad University School of Health and Welfare, Halmstad, Sweden; 8 Department of Health and Caring Sciences, Linneuniversitetet, Kalmar, Sweden

**Keywords:** COVID-19, qualitative research, organisational development

## Abstract

**Abstract:**

**Objective:**

To explore how primary healthcare workers in Sweden experienced and perceived the long-term impact of the pandemic on their work.

**Design:**

This is a descriptive qualitative study with individual semistructured interviews conducted 2 years after the onset of COVID-19. Data were analysed using an inductive thematic approach.

**Setting:**

Swedish primary healthcare units in rural and urban locations.

**Participants:**

29 healthcare providers (6 registered nurses, 7 assistant nurses, 8 physicians and 8 managers) in Swedish primary healthcare.

**Results:**

Data analysis yielded three overarching themes: (1) primary healthcare still affected by the pandemic; (2) primary healthcare changes made permanent; and (3) lessons learnt for handling future crises affecting primary healthcare. The participants experienced a high workload, even after the pandemic, and concluded that it would take years to catch up both mentally and workwise. Four lessons were learnt for future handling of crises that might affect primary healthcare: the importance of creating a cohesive primary healthcare management system to provide clarity regarding recommendations for how primary healthcare personnel should work, the need for management support at all levels, restricting and adapting the flow of information for primary healthcare and ascertaining the necessary resources if primary healthcare is to take on additional tasks.

**Conclusion:**

Two years after the onset of the COVID-19 pandemic, primary healthcare workers in Sweden experienced that their work was still affected by the pandemic. Our findings highlight the importance of ensuring sufficient recovery time and providing opportunities for reflection on the experiences of primary healthcare personnel. This also includes preparedness for managing the heavy workload and strained energy levels of healthcare workers in the aftermath of a crisis.

**STRENGTHS AND LIMITATIONS OF THIS STUDY**

- This study is based on repeat interviews and one limitation is that, of the 46 participants interviewed in 2020, twenty-nine agreed to be interviewed again for this study. Those who participated may be those who had the strongest opinions on the pandemic. They could be individuals with mostly negative experiences of the postpandemic period that they wanted to convey but they could also be those who believed that the pandemic was a learning period and that they succeeded in dealing with its challenges, and thus wanted to talk about this.
- Another limitation is that the pandemic was not officially declared over when the interviews were conducted, which could have affected the lack of time for reflection that many participants expressed.
- The results apply to primary healthcare in Sweden but need to be interpreted with caution for other healthcare systems and healthcare in other countries.
- The strengths include the multidisciplinary research team, which allowed for triangulation of the data to avoid a biased analysis; the rich dataset with participants from different professions; different geographical locations; and rural/urban primary healthcare units also increased the robustness of the results.

## Introduction

 The COVID-19 pandemic presented significant challenges to societies worldwide, with particularly detrimental effects on healthcare systems. The pandemic led to increased demand for testing, tracking and tracing capacities, the use of personal protective equipment and the scaling up of workforce capacities to manage the increased demand for care and overcrowded intensive care units.^1 2^ Media and research attention focused on hospital care, including intensive care units, but a large share of COVID-19-related care was managed by primary healthcare (PHC).^3 4^ PHC had a crucial role in reinforcing public health messages, differentiating patients with respiratory symptoms and early diagnostics of COVID-19, helping vulnerable people to cope with anxiety regarding the virus and reducing the demand for hospital services.^5 6^ PHC also had to manage the aftermath of the pandemic and handle the problems caused by reallocating resources to COVID-19-related care, which restricted access to a wide range of PHC services.^4^

PHC workers in Sweden had extensive responsibilities during the pandemic. The work tasks included triaging patients and identifying suspected cases of infection, coordinating with other healthcare providers and supplying holistic nursing practices in managing multiple infections simultaneously. PHC providers in Sweden also had major responsibility for the implementation of the COVID-19 vaccination programme as well as testing for COVID-19.^7^,^10^ Hence, the pandemic led to substantial changes in the work tasks and operations within PHC in Sweden.

The adaptations in PHC during the pandemic have been discussed regarding opinion-based lessons learnt^4 11 12^ and in empirical studies based on data from frontline providers or managers in countries such as Brazil,^13^ Benin,^14^ UK^15^ and Sweden.^7^,^9^ Furthermore, multinational studies^16 17^ have identified common challenges in PHC, such as managing information flows, and highlighted the differences in the approaches taken by PHC workers when dealing with patients with COVID-19 symptoms. However, the experiences of the pandemic among PHC providers and the impact on PHC operations in different countries may vary considerably depending on cultural and health system differences, emphasising the importance of national studies.

Despite numerous studies of how the pandemic affected PHC providers and operations, to our knowledge, no previous study has explored the late-pandemic experiences among PHC workers in Sweden. Therefore, 2 years after the onset of the pandemic but approximately 6 months before it was declared over in Sweden, this study aims to explore how PHC providers in Sweden experienced and perceived the impact of the pandemic on their work, that is, PHC operations. Some of the changes that were introduced in response to the pandemic could potentially be more effective than previous ways of working. Thus, it is important to document and analyse the lessons learnt by PHC providers for planning future PHC operations.

## Methods

### Study design and unit

We used a qualitative approach based on 29 semistructured interviews with PHC providers (managers, physicians, registered nurses and assistant nurses) from four regions in Sweden. To obtain rich data and improve trustworthiness, we reached out to six geographically different regions in Sweden in 2020, of which four agreed to participate. 17 PHC units participated in the study, encompassing nine urban and eight rural units. Two of the units were privately managed. Each unit had one to three individuals participating in the interviews (table 1). A qualitative approach was chosen due to the limited knowledge available regarding the impact of the late stage of the pandemic on the work environment in PHC. The most relevant method to gather information about this issue was deemed to be individual interviews to allow the participants to describe their unique experiences from the pandemic and its aftermath.

**Table 1:** Primary care unit demographics

Unit	Location	Participants	Public	Private
A	Rural	3	x	
B	Urban	1	x	
C	Urban	1	x	
D	Urban	2		x
E	Urban	1	x	
F	Rural	2	x	
G	Rural	1	x	
H	Rural	3	x	
I	Urban	1	x	
J	Urban	3	x	
K	Rural	3	x	
L	Rural	2	x	
M	Urban	1		x
N	Urban	1	x	
O	Urban	1	x	
P	Rural	1	x	
Q	Rural	1	x	

Rural: municipalities with a population of less than 15 000 inhabitants in the largest urban area. Urban: municipalities with equal to or more than 15 000 inhabitants in the largest urban area.

The Swedish healthcare system is divided into 21 regions. PHC units are either publicly or privately managed and mainly funded by taxes. The regions contract private PHC units, ensuring that out-of-pocket fees remain low because all citizens are insured by the state and have equal access to healthcare, regardless of the management of the healthcare centres. In 2019, there were 1140 PHC units in Sweden; of these, 44% were privately managed.^18^

PHC in Sweden encompasses a wide range of responsibilities, including the provision of basic healthcare services, treatments and diagnosis, as well as disease prevention measures for the entire population. It is the first line of healthcare to which citizens can turn to get healthcare.^19^ The regions’ PHC units cooperate with municipal healthcare regarding patients living in nursing homes or those with medical needs who are treated in their own homes. Municipal healthcare employs its own nurses and assistant nurses who can contact PHC units for access to physicians and nurses employed by the region.^20^

### Recruitment of participants

This is an interview study based on repeat interviews with the same individuals who participated in a previous study conducted by the same research group. The first interviews were conducted in autumn 2020 during the second wave of the pandemic, and the interviews for this study were conducted approximately 2 years later in autumn 2022. The study was thus carried out in the aftermath of the pandemic, with the focus on ‘consequences or after-effects of a significant unpleasant event’. The COVID-19 pandemic was formally declared over in Sweden in February 2023 when measures were abolished in Sweden. Details regarding the recruitment process are published elsewhere.^7^,^9^

For the current study, we emailed the participants (n=46) from the earlier studies,^7^,^9^ all of whom had approved renewed contact after 2 years, asking them if they would like to participate again. Of the 46 original participants, 29 accepted (table 1). Reasons for not participating were parental leave (n=1), undeliverable email (n=1), no answer or declined participation (n=12) and not showing up to the appointment (n=3). The reasons for declining participation were not specified.

Before conducting the interviews in 2020, the participants signed an informed consent form which stated that their confidentiality was guaranteed and that their full identity would not be known to anyone but the researchers. This informed consent from 2020 was formulated to be valid for this study as well.

Transcripts and audio files are stored, encrypted, at the file vault of Linköping University and no unauthorised persons have access to the data.

### Data collection

The semistructured interview guide developed for the 2020 study was revised by the authors to capture the participants’ experiences and perceptions pertaining to the period during the pandemic and the current situation. Questions included: ‘What changes were made in your PHC unit in response to the pandemic and what changes remained after the pandemic?’; ‘What are your experiences concerning what affected you the most, both now and then, during the pandemic?’; ‘How was your psychosocial work environment affected?’; ‘What lessons for a possible future crisis have you learned?’

The interviews were conducted by HF, KS, IS, JS and EK, all of whom have experience in conducting qualitative research. Before the interviews, the researchers re-read the participants’ transcripts from the interviews 2 years earlier to be able to discuss and compare the current situation. Each interview lasted between 21 and 57 min. All interviews were conducted via a digital application and recorded by audio and video. The audio file was labelled with a code and saved and the video file was deleted at the end of each interview. The interviews were performed between September and December 2022. All interviews were transcribed verbatim by an authorised transcription agency. The transcripts were then examined by HF and KS to ensure their accuracy of the transcripts.

### Data analysis

The interviews were analysed using an inductive thematic approach according to Braun and Clarke.^21^ Initially, all interviews were carefully read to gain a comprehensive understanding of the whole. Subsequently, HF and KS independently read and coded a selection of interviews, including one mutual interview, to ensure consistency in data interpretation. Following this step, HF, KS and MH met and discussed the early findings on two occasions, then read and coded the remaining interviews. HF, KS and MH collaborated to identify the main themes and subthemes throughout the coding process. After the initial coding and organisation into themes, HF, KS and MH met virtually to discuss the findings. The other researchers all read some of the interviews to get an overview and were then invited by email to discuss the interpretation, and consensus was reached after several face-to-face meetings with some of the group as well as written correspondence.

### Patient and public involvement

Not applicable, no patients involved.

## Results

The 29 participants interviewed for this study (table 2) worked in different PHC units in different professions, that is, managers, physicians, registered nurses and assistant nurses. The median age was 52 years and 86% of the participants were female. Analysis of the data yielded three overarching themes and nine subthemes (table 3).

**Table 2:** Participant characteristics

Characteristic	n (%)
Sex	
Male	4 (14)
Female	25 (86)
Profession	
Assistant nurse	6 (20)
Registered nurse	7 (24)
Physician	8 (28)
Manager	8 (28)
Age (years)	
37–47	9 (31)
48–58	16 (55)
59+	4 (14)

**Table 3:** Themes and subthemes

Themes	Subthemes
1. Primary healthcare work still affected by the pandemic	High workload
	Variable cooperation with other healthcare providers
	Lingering psychosocial impact
2. Primary healthcare changes made permanent	Separate infectious tracts
	Digital transformation
3. Lessons learnt for handling future crises affecting primary healthcare	Create a cohesive primary healthcare management system.
	Ensure that primary healthcare has management support at all levels.
	Restrict and adapt the flow of information for primary healthcare.
	Add the resources required for additional primary healthcare tasks.

### PHC work still affected by the pandemic

Although 2 years had passed since the onset of the pandemic, the participants’ daily work environment was still affected by the pandemic at the time of the interviews. Although the challenges were different from the most intense period of the pandemic, most participants described their work environment as still being very demanding. Factors contributing to this included persistent, or even increased, needs from patients as well as a shortage of healthcare workers, which influenced the daily work and work climate negatively. This theme consists of four subthemes.

#### High workload

Some participants described the current work situation as almost more demanding, with a higher workload than during the peak of the pandemic. The participants attributed the high workload to the need for care that had been postponed, including routine medical check-ups for chronic conditions such as type 2 diabetes and hypertension, and to patients’ impatience. Some participants mentioned that patients felt that their medical visits had been postponed during the pandemic and now had an urgent need for medical assistance. The participants described how the patients’ understanding and patience with delayed healthcare visits seemed to have run out and self-care advice was no longer sufficient. Some of the participants, in particular physicians and nurses, expressed disappointment that patients did not seem to have learnt that regular colds and short-term fevers heal themselves without medical care in a PHC unit. The participants commented that the phone queues to get in touch with PHC were long and many patients were not even able to get in touch with their PHC unit.

Patients were eager to get their annual physical check-ups, particularly because they relied primarily on blood samples to obtain new medications in the previous 2 years. According to participants, people now wanted to catch up on everything and assumed they should be at the top of the waiting list. Some patients express sentiments such as: ‘It’s simply a matter of hiring more healthcare workers; it’s not that complicated. Fit me in; it’s just a quick procedure.’

Essentially, their expectations have reverted to the way things were before the pandemic. During the pandemic, there was much greater understanding, but when the general public is no longer subject to restrictions, they struggle to comprehend that healthcare may still face challenges. (Assistant nurse 17)

One of my hopes was that people would learn to prioritize self-care, manage their expectations, and be patient, but unfortunately, that hasn’t been the case… (Physician 11)

Another explanation for the high workload was the shortage of healthcare workers in many PHC units. Some of the participants had colleagues who had quit their jobs due to the pandemic. Many of the participants expressed concerns regarding both the current situation and the future because they were experiencing a lack of physicians and registered nurses at their workplaces.

After all, we have also lost some healthcare workers in various professional categories. They couldn’t take it in the end and chose to change workplaces. I can see younger colleagues with less experience, they are more stressed about this. And that probably means that those of us who are a little more experienced stand firmly on the ground and are a little more seasoned, we try to support them and cover a little for them so that they don't hit the wall. We would like to keep them at work. But we still have some staff absences due to stress, especially this year. (Registered nurse 10)

Some participants felt the current situation was business as usual. They described the situation in the PHC as consistently stressful. Some mentioned that they appreciated that they were no longer forced to wear face masks all day, although they did so when they were in close contact with patients. Although stressful, the ‘normality’ of the current situation made it possible to do things together in the workplace as they had done before the pandemic, for example, days with education and development work.

I think it’s about the way it usually is, it’s always stressful [in primary healthcare]. (Physician 21)

My employees appreciate that things have returned to a sense of normality. We can attend educational events with the clinic and interact under different conditions compared with during the pandemic. We still maintain source control, meaning we wear a face mask when in close contact with patients, but we no longer need to wear it when walking in the corridors or in the break room. We've come to value what was once considered a normal condition [no face masks]. Employees often say ‘How nice it is that we don’t have to wear masks.’ (Manager 24)

#### Variable cooperation with other healthcare providers

Many participants noted that collaboration with secondary care was hindered due to the pandemic, citing factors such as unusually long queues for specialised care. The difficulty in accessing secondary care often resulted in an increased workload for healthcare personnel in PHC because the patients turned to them when they needed care.

For example, at the heart clinic, where they have long queues, a patient referred for echocardiography due to heart failure symptoms was returned to primary care with the response: Yes, it appears to be heart failure; treat it accordingly. We won’t contact the patient. (Physician 1)

After all, we have no one else to lean on and the patients have nowhere else to go, so it ends up on our lap. (Physician 9)

Experiences among the participants varied regarding cooperation with municipal healthcare. Some of the participants thought it had improved and was now working better than before the pandemic, but others did not share this view. A few participants found themselves dealing with their responsibilities at their primary care unit and shouldering some of the work typically assigned to municipal nurses.

It doesn’t seem like they [municipal nurses] have anyone to back them up. Sometimes I think there are such odd questions, I mean, they [municipal nurses] are also nurses, they shouldn’t call me to ask what they should do, they are the ones who see the patient. (Registered nurse 4)

#### Lingering psychosocial impact

The participants described how the pandemic had exhausted them and that they still experienced a great deal of fatigue 2 years after the onset. Some of the participants also described that they wanted to have more opportunities to reflect on their experiences, but time pressure never allowed for reflection. Some of the participants expressed a decline in work-related levels of energy, which emerged after the most acute phase of the pandemic. They attributed the lower energy level to not being able to rest and to the high demands on healthcare to catch up on the tremendous burden of care that had built up during the pandemic.

After the summer, when we emerged from the pandemic and I had had a holiday, I felt a significant dip in motivation, unlike anything I’ve experienced in my entire professional career. It may have been a result of the fatigue caused by all our battling during the pandemic. (Manager 29)

Despite the perceived fatigue and exhaustion, there was also a feeling of empowerment and strength expressed by some participants, having successfully navigated through the most intense period of the pandemic. This led participants to reflect on the possibilities that arise when colleagues support each other. Looking back on the most intense period of the pandemic, participants expressed a sense of wonder, asking themselves, ‘What have we actually been through?’

I believe we all feel a sense of empowerment, having successfully navigated this together and making it as manageable as we could, not just for ourselves but also for our patients. (Assistant nurse 17)

Despite some advantages in perceived social support among the participants 2 years after the pandemic began, some described that this level of heightened trust and empowerment within the workplace group had declined when things got back to a more business-as-usual situation.

In our work group, there are now more minor grievances compared with how it was during the peak of the pandemic when we simply didn’t have the time or energy for them. Things have returned somewhat to our normal routine, allowing us to become irritated by small matters. During the worst times, such things as trivial issues weren’t even on our radar; our focus was solely on survival and solidarity. (Registered nurse 10)

### PHC changes made permanent

The pandemic led to many operational changes in PHC, some of which became permanent. This theme comprises two subthemes, each describing distinct and lasting effects on the work environment and/or daily work that the participants attributed to the pandemic.

#### Separate infectious tracts

The establishment of distinct physical infectious pathways was highlighted as a lasting change in response to the pandemic. Regardless of the participant’s profession, all agreed that separate infectious tracts were a highly beneficial factor that came out of the pandemic. Looking back on how the patient flow had been before, many of the participants could not imagine going back to mixing patients with infections with other patients at the PHC, particularly not if the patients were old or fragile.

We still have dedicated infection rooms for patients with contagious illnesses. Instead of entering the health centre, there’s a separate room accessible from the outside. (Registered nurse 3)

Why would we have many sick individuals sit together in a waiting room, coughing and exposing each other to illness? It’s hard to grasp that now. I doubt we’ll return to such practices, with everyone using the same entrance. (Registered nurse 4)

#### Digital transformation

The pandemic significantly accelerated the digitalisation of work, including digital patient consultations, online meetings and remote training and education. The participants pointed out some benefits of this rapid, almost inevitable, transformation to increased digitalisation. Digitalisation made it possible to work from home and participate in various training and educational events without wasting valuable time on travelling. Many of the participants believed that the pandemic accelerated the digital transformation that had already begun at a slower pace before the pandemic. The pandemic resulted in both patients and healthcare workers having more positive attitudes towards the use of digital tools in PHC.

The digital ways of working, that’s probably what we mainly take with us. In cases of crises, we will be able to switch over quickly. But the digital way, that’s what it is all about, and you can work; everything doesn’t have to be the way it’s always been. You can work a little differently; that’s also possible. (Manager 22)

Despite the mostly positive experiences of digitalisation, some participants pointed out that the digital tools were not always user-friendly. For example, complex booking systems made it easier to simply contact the patient by a regular phone call.

We have some colleagues who put in more effort and tried using it, but they don’t think that a video appointment provides much additional information. It’s a bit complicated, at least for us, because it means double booking in two different calendars: one for video appointments and one for the regular one. This ends up causing more frustration, and sometimes it seems easier to just pick up the phone. (Physician 20)

### Lessons learnt for handling future crises affecting PHC

Some aspects of the pandemic yielded important insights to be considered in the case of future crises that might have an impact on PHC. This theme describes four types of lessons on how to act in the future as shared by the participants. The theme includes participants’ experiences of situations where different approaches might be considered in the future as well as participants’ preferences for how to handle a potential new crisis.

#### Create a cohesive PHC management system

The participants lamented that they did not have a national cohesive management system for PHC during the pandemic. This made it difficult to follow and adhere to recommendations on how they should work because there were disparate guidelines for different regions in Sweden. Furthermore, guidelines could even differ within regions depending on the PHC unit where the participants worked. This lack of coherence raised many questions and created considerable confusion, making it difficult to keep up with and recognise which guidelines to follow. In addition, the absence of standardised guidelines also left many patients confused and frustrated, making them contact their PHC unit with numerous questions. The participants described how they were often unable to answer the questions and provide clarity because they lacked information themselves.

I had several conversations with patients who questioned us ‘Why don’t you do this and why don’t you do that? They said this on the news, they said that in the daily newspaper.’ (Manager 23)

I think that you need to keep the governance more national and not regional because it will be too different. Having a guideline from the public health authority, one from the region and one from the specific health centre and so on, it doesn't work. (Physician 21)

Another aspect concerning the incoherent management system highlighted by the participants was the inadequate preparedness for the pandemic. There was an obvious shortage of personal protective equipment in several regions, leaving the healthcare workers with limited possibilities to protect themselves from infection. Participants emphasised the importance of being better prepared in the future and having a more appropriate inventory of protective equipment.

#### Ensure that PHC has management support at all levels

Nurses, assistant nurses and physicians emphasised the critical importance of supervisors supporting them in their work, especially during crises. Additionally, supervisors expressed a need for support from their counterparts in other units, such as through common meetings where they could discuss their activities, as well as support from higher level management. Many participants received good support from their closest manager. One of the registered nurses described the importance of structured and clear leadership, for example, having morning meetings to share oral information and updates regarding routines as well as words of encouragement from their manager. Another registered nurse underscored the importance of well-organised leadership during the pandemic, highlighting the need to guide employees in priorities and timing of their work tasks.

Even in a pandemic and disaster, you have to take care of your employees. I think it’s good if primary healthcare management embraces this for the future. (Registered nurse 10)

I believe the manager had genuine concerns about us not taking the right precautions, both for our own well-being and for the safety of the patients. They wanted to ensure that our work was carried out meticulously to prevent any chance of illness due to carelessness. Consequently, every morning, we diligently reviewed our routines and procedures, emphasizing precision. (Registered nurse 3)

One manager described how important it was to take care of the different needs of the employees, both personally and professionally. Another manager emphasised how crucial it was to get support from a group of managers from different PHC units in the region. In this region, regular digital meetings were held between managers from different PHC units. Although managers recognised the need for well-organised and structured leadership during the pandemic, this was not always possible to achieve based on rapid and unexpected changes. Often, decisions had to be made quickly, based on a limited amount of information, which caused a great deal of uncertainty for the managers.

There is such good dialogue and a good atmosphere in the large management group, so there has been great support. (Manager 26)

#### Restrict and adapt the flow of information for PHC

The participants were overwhelmed by the amount of information during the pandemic. They described that it was impossible to take in all the information. This was stressful and added to an already high workload. Many participants wished for less information, preferably limited, tailored high-quality information rather than overwhelming amounts of unstructured information, which they felt they received. According to the participants, the information received was not always tailored for PHC. Some of the participants underscored the importance of letting healthcare employees receive information before nationally televised press conferences were held. This would have made it easier to meet and respond to the patient’s questions and concerns, which usually arose in response to these press conferences.

I don’t know how it could have been handled better, but there were an incredible number of emails and various restrictions from day to day. And it was impossible to keep up to date. (Assistant nurse 14)

We need to consider how we can ensure that we have the necessary information before the patients. This was prompted by discussions among my colleagues. (Assistant nurse 7)

#### Add the resources required for additional PHC tasks

According to the participants, lack of resources added stress during the pandemic and increased the daily workload. Many managers and physicians experienced that PHC was overburdened and lacked additional resources to manage the new tasks imposed by the pandemic. Participants experienced that they were asked to perform many additional work tasks, which inevitably increased their level of stress and further limited opportunities for recovery. The participants noted that they did not receive any extra financial or workforce support from the region, despite the additional tasks imposed on PHC. With an eye towards future crises, the participants expressed a desire for the provision of appropriate prerequisites to accommodate additional responsibilities. They also called for greater understanding of the challenging operations of PHC among decision-makers.

More and more things are being transferred to primary care, but resources are not being added at the same rate, or not at all, I would say. (Registered nurse 8)

We might need to raise our concerns more forcefully to prevent misguided decisions from being made initially, especially when those decisions are coming from individuals or entities that lack a true understanding of what we do in primary healthcare. It doesn’t seem right for outsiders to dictate how we should operate or structure our organization. For instance, the way we organized procedures for sampling, contact tracing, and vaccinations was rather irrational. It should have been handled separately by an external organization, not within our primary healthcare system. (Manager 29)

There were many situations when secondary care faced challenges during the pandemic. Some participants believed that certain issues that should be the responsibility of specialty care were shifted to PHC. Despite having to perform non-PHC tasks no consideration was given to the situation in PHC, which was simply expected to help out.

We can’t define our mission as well as others can. We often hear statements like, ‘That clinic is currently overwhelmed and can’t handle this,’ and as a result, they transfer all the responsibilities for, for example, sick leave notes from [specialist clinic X] to primary healthcare. (Physician 20)

## Discussion

This study aimed to explore how PHC workers in Sweden experienced the work-related impact of the COVID-19 pandemic 2 years after the onset. In summary, the participants described a high workload and impatience from patients wanting medical attention after a long period of postponed appointments. The pandemic had resulted in a more cautious way to handle patients with infections using separate infection tracts as well as expedited implementation of digital solutions for patient consultations and collegial meetings. Four lessons were identified for future handling of crises: the importance of a national cohesive PHC management system to ensure uniform recommendations; the need for management support at all levels; restricting and adapting the flow of information for PHC; and ascertaining the resources required if PHC is to take on additional tasks.

Surprisingly, the work environment in PHC was regarded almost as challenging 2 years after as during the pandemic. In addition to the healthcare backlog due to the pandemic, there was a shortage of healthcare personnel, partly due to resignations after the pandemic. The risk that patients would delay addressing their health problems during the pandemic, leading to an increased future workload in PHC, was foreseen by Rawaf *et al*.^4^ However, in contrast to our findings, Rawaf *et al*^4^ thought it would be difficult to encourage people to seek healthcare again after the pandemic. Based on our interviews, patients wanted immediate medical attention when the pandemic slowed down which resulted in a high workload in PHC over a prolonged period.

Organisations tend to learn *through* rare events rather than *from* them (22, p 850). Rare events can make organisations and workers aware of structures and procedures that previously were taken for granted, thus making people more open-minded to changes.^22^

Our findings suggest that the accelerated process of digitalisation that took place during the pandemic was an example of ‘learning through an event’, both in terms of interaction with patients (ie, telemedicine) and meetings with colleagues. Although digitalisation was already in progress in Swedish PHC before the onset of the pandemic,^22^ the outbreak accelerated this shift significantly.^7 8 23^ Similarly, healthcare personnel worldwide have observed rapid adoption of digital working tools and a shift in attitudes towards digital healthcare among patients during the pandemic.^24 25^ Overall, participants in our interviews found digitalisation to be beneficial. It allowed for remote work when necessary and made it easier to attend meetings and educational events without the requirement for travel.

Numerous studies have emphasised the importance of learning from the pandemic to be prepared for future crises.^5 26^ Participants reflected on the lack of preparedness in their own PHC units and at other organisational levels within the PHC system at the beginning of pandemic. For example, there was a shortage of personal protective equipment at all levels, from individual PHC units to regional and central inventories. The need of increased supplies of personal protective equipment to enhance preparedness for future crises has also been pointed out by others.^7 14 27^ This lack of preparedness seemed global during the pandemic, revealing problems in maintaining global supply chains during crises.^28^ Our findings are consistent with research drawing attention to the importance of resilient healthcare system, that is, a system with the capacity to prepare for and uphold core functions during a crisis.^26^ A more resilient PHC requires collaboration with secondary care, which will ultimately also result in high-quality care during non-strained times.^27^ Several individual as well as organisational strategies have been identified to strengthen resilience among healthcare workers amidst the pandemic.^29^,^32^ These included the importance of supportive management at all levels, between PHC unit managers and healthcare personnel, as well as between higher level managers and PHC managers. Additionally, participants underscored the need for a cohesive national healthcare system with consistent recommendations and guidelines. Participants in our study emphasise that receiving and interpreting information during this time could be particularly stressful. The amount and different sources of information in the Swedish PHC could be attributed to the lack of cohesive national management. Similar experiences of short-notice new guidelines and the fact that patients were sometimes updated earlier than healthcare personnel have also been reported from other European countries during the pandemic.^27^ Accurate and updated information at the right time is important when handling crises to avoid rumours and misconceptions.^33^ Various solutions to the problem of information overload in PHC have been proposed. de Carvalho *et al*^34^ described how a new work task, an information broker, was created in PHC in Brazil to navigate the large amount of conflicting and occasionally inaccurate information. Receiving and interpreting information could be very stressful according to the participants in our study. Another solution for keeping the information succinct and comprehensible has been proposed by US researchers Poonia and Rajasekaran.^35^ They developed a quicksheet for healthcare personnel, consisting of one page with easily accessible information, which was sent out in emails; updating the information was the responsibility of a designated person.

Our participants described how PHC was assigned new tasks and given increased responsibilities for patient care without additional personnel resources during the pandemic. Some participants expressed that the workload and stress during the pandemic had consequences in terms of colleagues resigning and loss of energy in the workgroup. It has previously been shown that increased workload in healthcare organisations, particularly those already operating with limited resources, can lead to adverse outcomes such as increased staff turnover, compromised patient safety and decreased efficiency.^36^ Conversely, healthcare organisations with a certain degree of excess capacity appear resilient to these negative effects having resources to go beyond regular work tasks and helping colleagues. Periods of heightened intensity should ideally be followed by intervals of recovery and reflection.^36^ Unfortunately, such intervals were not evident in the experiences of the participants in our study, potentially leading to deleterious effects on work satisfaction and an increased likelihood of turnover.^36^

This study has a few limitations which should be taken into consideration. The participants’ interest in joining the study might have influenced the results. For instance, some may have had adverse pandemic experiences, prompting them to shed light on related issues, while others might have experienced positive changes they wanted to discuss. Despite these limitations, our study boasts significant strengths. We recruited participants from diverse regions, spanning both rural and urban PHC units. Our interviewees encompassed a wide spectrum of individuals, including men and women of various ages and professions, thereby enhancing the credibility of our research. Moreover, our research team is multidisciplinary, comprising resident physicians in PHC, postdoctoral researchers, a political scientist/sociologist, a public health scientist, a registered nurse and a behavioural economist. Our team members, belonging to different universities and institutions, contribute with diverse perspectives and experiences to the interpretation of our findings. Our findings underscore the critical importance of leveraging the lessons learnt from the COVID-19 pandemic to enhance healthcare systems, bolster support for healthcare workers and fortify readiness for future crises.

## Conclusion

In summary, the effect of the COVID pandemic was still apparent in PHC in Sweden 2 years after the pandemic onset. The workload was high with healthcare backlog to catch up and healthcare personnel described both loss of energy and proudness of what they had been through. Distinct infectious pathways for patients with infections and implementation of digitalisation were permanent changes described.

The experiences of PHC workers in the aftermath of the pandemic have reinforced the importance of building a resilient healthcare system with structured national guidelines as well as emphasising the importance of better preparedness for future crises. Our findings highlight the importance of sufficient recovery time and opportunities for reflection for PHC personnel. This also includes preparedness for managing the workload and strained energy levels of healthcare workers that the aftermath of a crisis entails.

## Data Availability

Data are available upon reasonable request.
