# Supplementary material for: Primary healthcare in the aftermath of the COVID-19 pandemic: a qualitative interview study in Sweden
Source: BMJ Open. 2024 Jul 27;14(7):e085527. doi: 10.1136/bmjopen-2024-085527 (PMC11284870; doi:10.1136/bmjopen-2024-085527)
Supplement: Supplementary file 1 [file bmjopen-2024-085527.reviewer_comments.pdf]

PEER REVIEW HISTORY

BMJ Open publishes all reviews undertaken for accepted manuscripts. Reviewers are asked to complete a checklist review form (**http://bmjopen.bmj.com/site/about/resources/checklist.pdf**) and are provided with free text boxes to elaborate on their assessment. These free text comments are reproduced below.

ARTICLE DETAILS

**TITLE (PROVISIONAL)**	Primary healthcare in the aftermath of the COVID-19 pandemic: a qualitative interview study in Sweden
**AUTHORS**	Fernemark, Hanna; Hårdstedt, Maria; Skagerström, Janna; Seing, Ida; Karlsson, Elin; Nilsen, Per; Schildmeijer, Kristina

VERSION 1 – REVIEW

**REVIEWER**	Wanat, Marta<br>Oxford University, Nuffield Department of Primary Care Health Sciences
**REVIEW RETURNED**	30-Apr-2024

**GENERAL COMMENTS**	Thank you for the opportunity to review this manuscript.<br>It addresses an interesting question of the long-term impact of the covid pandemic on primary care.<br>I have a number of comments which I would like authors to consider:<br><br>1. Abstract; the objective should perhaps be revised to highlight the long-term impact or in the aftermath of covid-19 pandemic; the conclusions are longer than the results which seem a bit unbalanced<br><br>2. Methods: 29 interviews are mentioned in the design but they should be mentioned (and are) much later on. The main query is whether it is a longitudinal study or not. It seems that it a follow up study to the first one. This is then brought up in the data collection in that previous interviews were consulted. However, the aim of this paper does not seem to compare T1 and T2 but rather explore what people ae thinking about the pandemic now.<br>Please also clarify how the initial consent covered them for this interview as well.<br>Please clarify how themes were created<br><br>3. Discussion; It would be great to provide more structure to the discussion including summary of findings; and implications as at the moment it is quite long and difficult to follow.

**REVIEWER**	Wright, Michael C<br>University of Technology Sydney, Health Economics Research and Evaluation
**REVIEW RETURNED**	07-May-2024

**GENERAL COMMENTS**	Thank you for the opportunity to review this paper reporting the results of qualitative interviews of general practices in Sweden

	conducted two years after a previous study which sampled the same general practices .<br><br>This is an interesting paper which highlights the ongoing impacts of the pandemic on the general practice workforce, business models and financial viability. I think this paper would be of interest to a broad primary care audience and reinforces some of the findings of the impact of the pandemic on general practices reported in the UK, USA and Great Britain.<br><br>The paper is well written, the methods are clear and I would recommend the paper's publication in BMJ open. The following comments are minor and relate to the abstract, methods/sampling and the results (detailed below). Dealing with these queries will hopefully assist the flow of the paper. (I would not see any of these issues impacting publication.)<br><br><br>Abstract:<br><br>• Line 23.<br>"The participants experienced a high workload, even after the pandemic, and concluded that it will take years to catch up both mentally and workwise"<br>Does this imply that the participants have a greater workload than prior to the pandemic and they do still not have the capacity to manage this ongoing increased workload? If that is the case, will they ever 'catch up'?<br><br>Methods/sample:<br><br>• Was each interview participant from different PHC units or were there multiple interviewees from the same PHC unit?<br><br>• Sample demographics<br>o Was information collected about the PHC clinics where respondents worked?<br>o Where were they located (rural or urban) and were they public or private?<br><br>• Sample is described as Interviews with PHC providers from four regions in Sweden. Why were these four regions chosen and is there any information about their urban or rural location?<br><br><br>Results:<br><br>• P 11 High workload- the data presented suggest that it was not just the quantity of the workload, but also a change in the nature of work – with increased stress from patients being less understanding about PHC until capacity- and loss of staff contributing to a sense of more work<br><br>• The authors describe 'strained cooperation with other healthcare providers' as a sub-theme - the data provided suggest lack of capacity, and then page 14 line 16 reports variable cooperation so not sure of the consistency of this sub-theme- (perhaps 'variable cooperation' might be better description)<br><br>• P17 line 50

	coherent primary healthcare management system- could the authors clarify whether this needed at a regional or national level (or both)?<br>(repeated in conclusion page 25 line 37<br><br>• P 18 line 46<br>Ascertain that primary healthcare has management support at all levels<br>Could the authors clarify the meaning of this finding? What are 'at all levels' – should this be 'ensure 'rather then 'ascertain'?<br><br><br>Thank you for the opportunity to review this paper.

VERSION 1 – AUTHOR RESPONSE

Reviewer: 1
Dr. Marta Wanat, Oxford University
Comments to the Author:

Thank you for the opportunity to review this manuscript.
It addresses an interesting question of the long-term impact of the covid pandemic on primary care.

I have a number of comments which I would like authors to consider:

1. Abstract; the objective should perhaps be revised to highlight the long-term impact or in the aftermath of covid-19 pandemic; the conclusions are longer than the results which seem a bit unbalanced.
Agreed and revised, please see abstract row 18 for clarification. Regarding the length of the conclusion compared with the results, we agree with your comment and have revised it accordingly. Please see abstract, rows 26-34.

2. Methods: 29 interviews are mentioned in the design but they should be mentioned (and are) much later on. The main query is whether it is a longitudinal study or not. It seems that it a follow up study to the first one. This is then brought up in the data collection in that previous interviews were consulted. However, the aim of this paper does not seem to compare T1 and T2 but rather explore what people ae thinking about the pandemic now.
We have discussed this extensively and concluded that the current study is not a longitudinal study, as we neither evaluate an implemented intervention nor compare earlier interview data with current data in the analysis. Therefore, we decided to label the interviews as 'repeated' interviews. We re-read the previous interviews from 2020 to catch up and familiarize ourselves with the participants before conducting the new interviews.

Please also clarify how the initial consent covered them for this interview as well.
We have now clarified this in the text. Please see page 8, row 143-144.

Please clarify how themes were created
Thank you for noticing our mistake with labeling in the data analysis section. We have, incorrectly, labelled themes and sub-themes as categories and sub-categories respectively. This is now corrected, please see data analysis section for clarity. Page 9. Rows 172-173.

3. Discussion; It would be great to provide more structure to the discussion including summary of findings; and implications as at the moment it is quite long and difficult to follow.
Thank you for your comment. This measure will improve the manuscript. Following your suggestion, we have undertaken a major revision of the discussion. We emphasized the main findings and discussed them in fewer, more cohesive sections. As a result, the discussion and conclusion were

shortened by approximately 300 words. Nevertheless, with the added section on ‘strengths and limitations’ in the discussion section, the word count is approximately the same as earlier.

Reviewer 2:
Dr. Michael C Wright, University of Technology Sydney

Thank you for the opportunity to review this paper reporting the results of qualitative interviews of general practices in Sweden conducted two years after a previous study which sampled the same general practices . This is an interesting paper which highlights the ongoing impacts of the pandemic on the general practice workforce, business models and financial viability. I think this paper would be of interest to a broad primary care audience and reinforces some of the findings of the impact of the pandemic on general practices reported in the UK, USA and Great Britain. The paper is well written, the methods are clear and I would recommend the paper’s publication in BMJ open.

The following comments are minor and relate to the abstract, methods/sampling and the results (detailed below). Dealing with these queries will hopefully assist the flow of the paper. (I would not see any of these issues impacting publication.)

Abstract:
• Line 23. “The participants experienced a high workload, even after the pandemic, and concluded that it will take years to catch up both mentally and workwise” Does this imply that the participants have a greater workload than prior to the pandemic and they do still not have the capacity to manage this ongoing increased workload? If that is the case, will they ever ‘catch up’?
This finding is a bit surprising, isn’t it? We also share the concern about whether they will ever catch up. Our impression from conducting the interviews is that participants hoped to 'catch up' but struggled to see when this might happen due to the continuous high workload in primary healthcare.

Methods/sample:
• Was each interview participant from different PHC units or were there multiple interviewees from the same PHC unit?
We have clarified this in the methods section, please see the revised manuscript, page 5, row 104 and new table 1.

• Sample demographics o Was information collected about the PHC clinics where respondents worked? o Where were they located (rural or urban) and were they public or private?
Thank you for this important remark. This is now added in a new table, table 1. Please see page 5, rows 101-105 and pages 6-7 (new table 1).

• Sample is described as Interviews with PHC providers from four regions in Sweden. Why were these four regions chosen and is there any information about their urban or rural location?
This is now clarified under the section study design and unit, page 5, rows 101-105.

Results:
• P 11 High workload- the data presented suggest that it was not just the quantity of the workload, but also a change in the nature of work – with increased stress from patients being less understanding about PHC until capacity- and loss of staff contributing to a sense of more work
This aligns with our interpretation of the results. The subtheme encompasses various aspects of the term 'workload'.

• The authors describe ‘strained cooperation with other healthcare providers’ as a subtheme - the data provided suggest lack of capacity, and then page 14 line 16 reports variable cooperation so not sure of the consistency of this sub-theme- (perhaps ‘variable cooperation’ might be better description)
This is a good remark, we struggled a bit to find the right title but we agree upon your suggestion and have now changed it accordingly.

• P17 line 50 coherent primary healthcare management system- could the authors clarify whether this needed at a regional or national level (or both)? (repeated in conclusion page 25 line 37

This is now clarified in the text, thank you for paying attention to this. Please see page 17, row 339 and page 25, rows 549-550.

• P 18 line 46 Ascertain that primary healthcare has management support at all levels Could the authors clarify the meaning of this finding? What are ‘at all levels’ – should this be ‘ensure ‘rather then ‘ascertain’?
As you point out, ‘All levels’ does not explain itself; we have now tried to clarify this in the manuscript. Also, ensure is, as suggested, a more suitable way of expressing the subcategory. Please se page 18, rows 361-366.

Thank you for the opportunity to review this paper

VERSION 2 – REVIEW

**REVIEWER**	Wanat, Marta<br>Oxford University, Nuffield Department of Primary Care Health Sciences
**REVIEW RETURNED**	08-Jul-2024

**GENERAL COMMENTS**	Thank you for the revision and addressing the comments. I am happy with the revision.
